# Diagnosis and management of invasive candidiasis in critically ill patients: SIAARTI multidisciplinary statement

**DOI:** 10.1186/s44158-025-00299-y

**Published:** 2025-12-02

**Authors:** Andrea Cortegiani, Gennaro De Pascale, Giulia De Angelis, Marco Falcone, Arianna Ferrini, Arianna Forniti, Milo Gatti, Massimo Girardis, Giacomo Grasselli, Federico Pea, Matteo Rinaldi, Maurizio Sanguinetti, Pierluigi Viale, Antonino Giarratano

**Affiliations:** 1 Department of Precision Medicine in Medical, Surgical, and Critical Care, University of Palermo, (Me.Pre.C.C.), Via del Vespro 129, Palermo, 90127 Italy; 2 General Intensive Care Unit, University Hospital Policlinico “Paolo Giaccone”, Palermo, Italy; 3 Dipartimento di Scienze biotecnologiche di base, cliniche intensivologiche e perioperatorie, Università Cattolica del Sacro Cuore, Roma, Italy; 4 Department of Emergency, Anaesthesia, and Resuscitation Sciences, Università Cattolica del Sacro Cuore, Fondazione Policlinico Universitario A. Gemelli IRCCS, Rome, Italy; 5 Dipartimento di Scienze di Laboratorio ed Ematologiche, Fondazione Policlinico Universitario A. Gemelli IRCCS, Roma, Italy; 6 Department of Clinical and Experimental Medicine, Azienda Ospedaliero Universitaria Pisana, Pisa, Italy; 7 Frelance, London, UK; 8 Department of Medical and Surgical Sciences, Alma Mater Studiorum University of Bologna, Bologna, Italy; 9 SSD Clinical Pharmacology, IRCCS Azienda Ospedaliero-Universitaria Di Bologna, Bologna, Italy; 10 Department of Anaesthesia and Intensive Care, University Hospital of Modena, University of Modena and Reggio Emilia, Modena, Italy; 11 Department of Emergency and Urgent Care, Fondazione IRCCS Ca’ Granda Ospedale Maggiore Policlinico, Milan, Italy; 12 Department of Pathophysiology and Transplantation, University of Milan, Milan, Italy; 13 Department of Integrated Infectious Risk Management, IRCCS Azienda Ospedaliero-Universitaria Di Bologna, Bologna, Italy; 14 Department of Anaesthesia, Intensive Care and Emergency, University Hospital Policlinico Paolo Giaccone, Palermo, Italy

**Keywords:** Candidemia, Invasive candidiasis, Antifungal, Intensive care unit

## Abstract

Critically ill patients in Intensive Care Units are at high risk of developing invasive candidiasis (IC). Delay in diagnosis and suboptimal management contribute to high mortality rates, highlighting the need for an appropriate and patient-tailored approach. The Italian Society of Anaesthesia, Analgesia, Resuscitation, and Intensive Care (SIAARTI) convened a multidisciplinary panel, involving intensivists, infectious disease specialists, microbiologists, and pharmacologists, to develop consensus-based statements on the diagnosis and management of IC in critically ill patients. The panel formulated 13 statements addressing key aspects of care, including identification of major risk factors for IC, the role of biomarkers to support therapeutic decision-making, and optimal selection of antifungal agents based on pharmacokinetic/pharmacodynamic (PK/PD) considerations and site-specific characteristics. The document also provides guidance on the use of therapeutic drug monitoring (TDM) to optimize antifungal efficacy. Special attention is given to infections in specific anatomical sites, such as the peritoneal cavity, central nervous system, eye, and biofilm-associated infections, where drug penetration and activity must be carefully considered. This multidisciplinary statement offers a practical framework aimed at improving clinical decision-making for the management of invasive candidiasis in critically ill patients.

**Supplementary Information:**

The online version contains supplementary material available at 10.1186/s44158-025-00299-y.

## Background

Critically ill patients admitted to Intensive Care Units (ICUs) are at high risk of developing invasive infections caused by Candida spp. [1]. Invasive candidiasis (IC), including candidemia and deep-seated candidiasis, represents a significant clinical challenge, characterised by high mortality rates, particularly in patients with risk factors such as prolonged central venous catheter use, broad-spectrum antibiotic therapy, parenteral nutrition, and immunosuppression, major abdominal surgery [2, 3]. The diagnosis of these infections is often delayed, and empirical treatment may be inadequate, highlighting the need for a targeted diagnostic and therapeutic approach to improve patient outcomes [4].

The pathogenesis of invasive candidiasis is complex, influenced by both host-specific factors and the interactions of these factors with medical interventions, such as invasive devices and intensive care therapies required for organ support. Clinical manifestations vary depending on the anatomical site and severity of the infection, making early and accurate diagnosis crucial but challenging, especially for intrabdominal candidiasis, the most common form of deep-seated candidiasis [5].

IC may involve several anatomical sites beyond the bloodstream, leading to specific diagnostic and therapeutic challenges. Intra-abdominal candidiasis is the most common form of deep-seated IC in critically ill patients, often complicating abdominal surgery, especially in case of anastomotic leaks, or recurrent perforations. Candida endocarditis, although rare, is associated with high mortality and usually requires both antifungal therapy and surgical management. Central nervous system (CNS) involvement (meningitis or meningoencephalitis) and ocular complications (endophthalmitis) represent anatomical sites where drug penetration is limited and therapeutic strategies must be carefully selected according to specific PK/PD characteristics. Urinary tract involvement may occur particularly in patients with indwelling catheters, while device-associated infections (prosthetic material, intravascular catheters) are often complicated by biofilm formation. In patients with candidemia, complementary exams such as ophthalmologic evaluation and echocardiography should be performed, particularly in those with persistent candidemia and/or clinical signs suggestive of metastatic infection, in immunocompromised patients, and/or in those with lack of definitive source control [4, 6]. Indeed, not performing these complimentary exams has been proven to be associated to higher risk of mortality in patient with candidemia, probably reflecting a lower quality of overall care [7]. A tailored diagnostic work-up is therefore essential to guide appropriate antifungal selection and to promptly identify complications of candidemia.

Indeed, the availability of effective antifungal therapies, including echinocandins and liposomal amphotericin B, has significantly advanced the management of IC. However, selecting the appropriate antifungal agent requires a thorough understanding of drug pharmacology, resistance profiles of isolated species, and patient’s clinical condition [3, 8]. Strategies that incorporate PK/PD monitoring and ensure the targeted use of resources are key to optimising clinical outcomes.

The Italian Society of Anaesthesia, Analgesia, Resuscitation, and Intensive Care (SIAARTI) convened a multidisciplinary panel, involving intensivists, infectious disease specialists, microbiologists and pharmacologists, to develop consensus-based statements on the diagnosis and management of IC in critically ill patients. This consensus multidisciplinary statement offers a practical framework aimed at improving clinical decision-making for the diagnosis and management of IC in critically ill patients.

## Methods

The SIAARTI board of directors selected the panelists based on their proven clinical and scientific expertise on the topic. This project was coordinated by an anesthesiologists-intensivist with expertise in methodologists (AC). It was not designed around PICO questions as consensus-based document. The process for defining the items was as follows: all panelists were invited to propose a list of topics considered relevant for discussion. The coordinator then consolidated these proposals into a single list, which was subjected to a structured blind voting process. Each panelist scored the items on a 1–9 scale (1 = no priority; 9 = critical). Items ranked as “critical” were selected and included in the document. This approach ensured that the content was derived from the collective judgment of the panel and reflected the highest-priority issues.

The items were assigned to one or more panel members according to their specific expertise, with the aim to develop statements and supporting rationales in the form of explanatory text based on their clinical and scientific experience.

This project did not involve a systematic literature review. However, each panel member was given the opportunity to propose relevant reference articles, which were included in the rationales.

The drafted statements and rationales were subjected to a consensus vote using a modified Delphi method, according to a pre-planned methodology. Each panel member was asked to assess the appropriateness of each statement using a 9-point Likert-like scale:

- 1–3: Inappropriate.
- 4–6: Uncertain.
- 7–9: Appropriate.

The pre-defined consensus criteria required the following:

- At least 75% of voters assign scores within one of the three ranges (1–3, 4–6, or 7–9).
- The median score falls within the same range.

During the first round of voting, panel members could provide anonymous comments and suggestions for modifying or rephrasing the statements. Since all statements reached a consensus during the first round, a second voting round was unnecessary.

The voting results are presented in tabulated form alongside the approved statements and rationales to ensure transparency and reproducibility of the process (Additional File 1).

Then, a manuscript draft was prepared by the methodologists and circulated among the other panelists. The final version was submitted for external review by an intensivist with expertise in infections in critically ill patients. The proposed modifications were discussed by the panelists and incorporated into the final version of the manuscript.

## Results

### Major risk factors for developing invasive candidiasis

1. Risk factors for invasive Candida infections are non-specific and common among most critically ill patients in Intensive Care Units.
2. Risk factors can be used as part of an overall clinical assessment to determine the appropriateness of initiating empirical antifungal therapy in suspected invasive candidiasis.
3. Critically ill patients with high risk factors, such as previous use of broad-spectrum antibiotics, central venous catheters, total parenteral nutrition, repeated and complicated abdominal surgery, and immunodepressed status, should be considered at high risk. Colonization by Candida spp. is an important risk factor and requires careful monitoring and management.

Most critically ill patients admitted to ICUs are at risk of developing invasive Candida infections [2, 3, 9]. This is supported by the presence of largely non-specific risk factors related to comorbidities, treatments, consequences of therapies or underlying disease, and/or the devices required for organ support. A recent meta-analysis identified 29 risk factors from 34 studies, encompassing demographic factors, treatments received during hospitalisation, and comorbidities [2]. Among these, neutropenia, HIV, and multisite Candida colonization had the most significant impact. Regarding therapeutic interventions, the presence of a central venous catheter (OR 4.7; 95% CI 2.7–8.1); broad-spectrum antibiotic therapy (OR 5.6; 95% CI 3.6–8.8); transfusions (OR 4.9; 95% CI 1.5–16.3); total parenteral nutrition (OR 4.6; 95% CI 3.3–6.3); and Candida colonization (OR 4.7; 95% CI 1.6–14.3) were the strongest associations in the final statistical model. Interestingly, demographic factors were not significantly associated with the risk of Candida infections. Furthermore, significant collinearity between risk factors highlighted their interdependence, making it difficult to isolate the true independent association of each factor, especially in critically ill patients.

Candida spp. colonization is a particularly critical risk factor that requires special attention because it represents a marker of the interaction between the host and the microorganisms. A recent meta-analysis found that ICU patients with Candida colonization have a significantly increased risk of developing invasive candidiasis (OR 3.32; 95% CI 1.68–6.58) compared to non-colonized patients [10].

Another recent meta-analysis found that the average ICU stay at the onset of candidemia was 12.9 days (95% CI 11.7–14.2) [9]. It is reasonable to consider that the cumulative impact of these risk factors increases with the length of ICU stay, as the pathophysiological process leading to invasive candidiasis has time to develop and progress (e.g., central venous catheter insertion, colonization, candidemia) [9, 11]. Regarding intra-abdominal candidiasis, a recent retrospective study including patients from 26 ICUs in European hospitals identified recurrent intestinal perforation (OR 13.90; 95% CI 2.65–72.82); anastomotic dehiscence (OR 6.61; 95% CI 1.98–21.99); the presence of abdominal drainage (OR 6.58; 95% CI 1.73–25.06); antifungal treatment (OR 4.26; 95% CI 1.04–17.46); or antibiotic treatment for seven or more days (OR 3.78; 95% CI 1.32–10.52) as independently associated with the development of intra-abdominal candidiasis [12].

## When is the initiation of empirical therapy indicated in suspected invasive candidiasis?

### Empirical antifungal therapy should be promptly considered in high-risk ICU patients with suspected invasive candidiasis to improve clinical outcomes and prevent infection progression

The appropriateness of non-targeted antifungal strategies in critically ill patients—i.e. the administration of antifungal agents before a definitive microbiological diagnosis—is a long-debated topic. The use of empirical antifungal therapy in critically ill ICU patients is widely debated due to the lack of clear evidence supporting a survival benefit [13]. Specifically, the cost–benefit balance of initiating empirical antifungal therapy (i.e. treatment in patients presenting with non-specific signs and symptoms of infection despite antibiotic therapy) in critically ill patients remains controversial despite decades of research [13, 14].

Two major randomised controlled trials [15, 16] indicate that initiating empirical antifungal therapy alone does not improve survival. Moreover, there is currently no strong evidence demonstrating a consistent mortality reduction with early empirical therapy [13]. However, evidence is heterogenous in terms of patient populations, settings, necessity of source control, and overall management, thus an overall benefit cannot be excluded [17]. Thus, there is a discrepancy between the association between early initiation of therapy and mortality in observational studies [18–20] and the findings of randomized clinical trials, and this underlines the difficult to define an appropriate overall strategy [21].

The 2021 Surviving Sepsis Campaign guidelines identified additional risk factors beyond those already described, including immunosuppression; neutropenia; clinical severity (e.g., high APACHE score); prior surgery; emergency gastrointestinal and hepatobiliary surgery; acute kidney failure; and dialysis [22]. These guidelines recommended that the initiation of empirical antifungal therapy should be based on risk factor assessment. Specifically, antifungal treatment is suggested for patients with sepsis or septic shock who are at high risk for fungal infections, while it is not recommended for those at low risk [22]. A task force from ESICM/ESCMID recommends initiating empirical antifungal therapy in patients with septic shock, multi-organ failure, and confirmed Candida colonization in at least one extra-digestive site (strong recommendation, low level of evidence) [23].

The latest clinical practice guidelines by the American Thoracic Society suggests against routine prophylactic or empiric antifungal agents against Candida spp. In critically ill patients without neutropenia or a history of transplant [24]. The recent global guidelines on Candida infections by the European Confederation of Medical Mycology (ECMM) and International Society of Human and Animal Mycology (ISHAM) moderately recommend starting empiric antifungal treatment for patients with septic shock or patients with deteriorating conditions with additional risk factors for candidemia, such as prolonged ICU stay, indwelling vascular catheter and Candida spp. colonization [4].

In this context, risk factor assessment, alongside the evaluation of clinical severity, is a key determinant in the decision to start empirical antifungal therapy in suspected invasive candidiasis, ensuring an appropriate evaluation of the cost–benefit ratio. A comprehensive case-by case clinical decision between initiation of empiric antifungal treatment versus watchful waiting and eventual timely initiation of targeted antifungal therapy seems to be the most reasonable approach to balance risks and benefit [25].

### The clinical value of the beta-D-glucan (BDG) test for initiating empirical antifungal therapy is limited to cases with very high BDG levels, and its use as a standard guide for all ICU patients requires further studies

The beta-D-glucan (BDG) test has been proposed as a useful tool to guide the initiation of empirical antifungal therapy, as it detects components of the Candida spp. cell wall, indicating potential invasive infection. A study by Christner et al. [26] evaluated the effectiveness of BDG testing in guiding therapeutic decisions for high-risk, critically ill patients. The results showed that BDG testing has moderate sensitivity (74%) and low specificity (45%) when using standard thresholds. However, very high BDG levels improved the test’s predictive ability, suggesting that BDG may help identify patients who could benefit from early antifungal therapy. The multicentre CandiSep trial [27] further investigated the use of BDG testing to guide antifungal therapy in sepsis patients at high risk of invasive candidiasis. While BDG testing enabled earlier initiation of antifungal therapy (median of 1.1 days in the BDG group vs. 4.4 days in the control group, *p* < 0.01), it did not lead to a significant reduction in 28-day mortality (33.7% in the BDG group vs. 30.5% in the control group; *p* = 0.53). Additionally, BDG testing was not shown to be cost-effective and associated to over-treatment since a significantly higher proportion of patients in the BDG group received antifungal within 96 h versus the control group (48.8% vs. 6%). The Candi-NET study reaffirmed that no robust data directly correlate high BDG levels with an increased risk of developing the disease. On the other hand, at least two studies have confirmed the high negative predictive value of BDG testing, making it particularly useful for early discontinuation of antifungal therapy in critically ill patients, especially in cases of negative test results [28, 29]. This suggests that while BDG testing may accelerate treatment initiation, it does not necessarily improve clinical outcomes unless combined with other risk factors and clinical indicators and may carry the risk of over-treatment. The high negative predictive value of BDG is a key factor, and its use as a tool for early discontinuation of antifungal therapy could provide significant clinical advantages. Specifically, in patients with septic shock or suspected fungal infection, a negative BDG test can help reduce unnecessary treatments and minimise the risk of antifungal-related adverse events [30, 31]. Conversely, in ICU settings, a reduction in BDG serum concentration appears to correlate with lower mortality. Notably, a > 70% BDG reduction has been associated with increased survival, with a specificity and positive predictive value of 100% [32]. The ECMM/ISHAM Candida global guidelines for diagnosis and management of candidiasis reports that diagnosis of IC should not be solely based on BDG testing and positive serum BDG alone is not recommended for initiating antifungal treatment. Indeed they moderately support the use of BDG testing to discontinue empirical antifungal treatment [4].

## Selection of antifungal agents based on the site and severity of infection

### In patients with sepsis and septic shock, empirical or targeted antifungal treatment should be based on broad-spectrum, highly fungicidal agents (i.e. echinocandins and L-AmB). After clinical stabilisation and confirmation of in vitro susceptibility, de-escalation to azoles is appropriate.

The appropriateness of antifungal therapy, even in its empirical phase, is essential to optimize the treatment of invasive candidiasis in critically ill patients. Numerous observational studies have demonstrated that, in addition to controlling the infectious source, selecting the appropriate antifungal agent is a key factor in improving the prognosis of patients with severe infections [12]. It is well established that risk factors for invasive Candida infections are multiple and non-specific, including prolonged ICU hospitalisation, broad-spectrum antibiotic therapy, immunosuppression, and complex abdominal surgery [13]. As a result, most antifungal therapies for Candida infections are initiated in patients without a documented infection, contributing to widespread antifungal use and increasing ecological pressure. This is evidenced by the rising prevalence of fluconazole-resistant Candida non-albicans species [33]. For these reasons, current international guidelines recommend the use of echinocandins (caspofungin, anidulafungin, micafungin, or rezafungin) as first-line therapy in critically ill patients with suspected or confirmed invasive candidiasis, followed by L-AmB as second-line, particularly in patients who have already been treated or who have risk factors for Candida non-albicans species resistant to echinocandins [4, 23, 34]. Unlike azoles, which exhibit time-dependent pharmacological activity and a fungistatic in vitro effect, echinocandins and L-AmB share concentration-dependent activity and a potent fungicidal action, allowing for rapid fungal load reduction [35]. A literature review including seven randomised studies with 1915 patients with candidemia and invasive candidiasis showed that echinocandin therapy was associated with reduced mortality (OR 0.65; 95% CI 0.45–0.94) and a higher clinical cure rate (OR 2.33; 95% CI 1.27–4.35) [36]. Conversely, while amphotericin B has the highest fungicidal activity and a prolonged post-fungal effect, it is still considered a second-line option due to the high toxicity risk of its original formulation (amphotericin B deoxycholate). However, a literature review involving 2352 patients found no superiority of echinocandins over amphotericin B as a first-line treatment. Instead, the liposomal formulation of amphotericin B was associated with a significantly lower risk of nephrotoxicity [36]. In critically ill patients, azoles should note be considered first-line agents for IC. Their role is mainly limited to step-down once clinical stability is achieved and susceptibility to common azoles is confirmed. This approach is both ecologically responsible and clinically safe in terms of efficacy and adverse events [37]. In settings without routine access to susceptibility testing, local epidemiology and resistance pattern should be taken into account before considering de-escalation to azoles. The ECMM-ISHAM global guidelines on IC marginally recommend fluconazole and voriconazole as second-line treatment for candidemia for the high-risk of treatment failure, the increased antifungal resistance (i.e. azole-resistance C. parapsilosis, C. auris, C. glabrata), drug-to-drug interaction and the need for TDM [4]. The same guidelines moderately recommend switching to an oral azole (fluconazole or voriconazole) after 5 or more days of echinocandin treatment in patients with haemodynamic stability, documented clearance of Candida from the bloodstream, absence of neutropenia, completed source control, ability to tolerate oral azole treatment and susceptibility confirmed to selected azoles [4]. For infections involving selected anatomical sites (e.g. CNS or ocular infections) their favourable tissue penetration may make them a suitable therapeutic option.

### In the treatment of invasive candidiasis at sites involving where antifungal penetration is limited (e.g. peritonitis, endophthalmitis, meningoencephalitis, cystopyelitis, and device-related infections), the choice of therapy should be guided by the drug’s ability to reach effective concentration at the site of infection (i.e. azoles and L-AmB) and anti-biofilm activity (i.e., echinocandins and L-AmB)

Another critical factor in treating invasive candidiasis is the site of infection and the tissue concentration profile of different antifungal agents. In patients with primary candidemia or Candida-associated infections where the device has been removed, the pharmacokinetic and pharmacodynamic (PK/PD) profile of echinocandins supports their role as first-line agents, given their high safety profile and reliable achievement of PK/PD targets, even in patients with septic shock or multi-organ failure.

However, several anatomical sites remain difficult to penetrate because of the hydrophilic nature of echinocandins and their high plasma protein binding, which limit tissue distribution. Current guidelines recommend azoles or polyenes as the only therapeutic alternatives for infections involving the CNS, ocular compartment, pericardial/pleural serous cavities, osteoarticular system, and urinary tract, depending on disease severity, susceptibility profile of the isolates, and the risk of adverse events [4].

A particular consideration is intra-abdominal candidiasis, one of the most common forms of invasive Candida infection. In this setting, fluconazole remains an appropriate choice for clinically stable patients with fully susceptible fungal isolates. However, in critically ill patients or cases with a high risk of azole resistance, the use of echinocandins as first-line therapy has been questioned [37]. Emerging pharmacokinetic studies has shown that caspofungin, anidulafungin, and micafungin achieve only ~ 15–30% penetration rate into peritoneal fluid, raising concerns about treatment failure and resistance selection, particularly with prolonged therapy [38]. To address this, strategies include the following:

- Increasing the daily dose of echinocandins (e.g. caspofungin 2 mg/kg loading dose, followed by 1.25 mg/kg/day), or
- Preferring L-AmB (3–5 mg/kg/day), which offers better pharmacokinetic properties due to its liposomal formulation [35].

For device-associated infections (e.g. endocarditis, prosthetic infections, or synthetic material infections), the presence of biofilm limits the effectiveness of several antifungals. Echinocandin are fungicidal against planktonic Candida spp. and retain activity against sessile form in biofilms while lipososomal amphotericin B (L-AmB) is active against both planktonic and sessile form. Azoles generally have limited activity against biofilm. Consequently, when biofilm Is suspected, an echinocandin or L-AmB are appropriate options in addition to prompt device removal when feasible [8]. Finally, except in rare cases of profoundly immunosuppressed patients with histological evidence of tissue invasion, the isolation of Candida spp. from respiratory secretions should be considered colonization and does not require antifungal treatment [39].

The emergence of resistant Candida species poses a major challenge in the management of invasive candidiasis in critically ill patients [4, 33]. Among these, C. auris has gained increasing relevance in ICU settings, being responsible for nosocomial outbreaks due to its ability to persist in the environment, its high transmission potential, and its frequent multidrug-resistant profile [40, 41]. Echinocandins are generally considered the first-line agents when C. auris is suspected or confirmed, although reduced susceptibility and resistance have also been described [4]. In addition, fluconazole resistance has become a growing concern in several countries, including in Europe. In a recent multicentre observational study, fluconazole resistance was found in 17% of C. parapsilosis isolates (from Italy, Turkey, and Greece) and 12% of C. glabrata isolates (from six European countries) [42]. This resistance significantly limits the use of fluconazole as a step-down agent and highlights the importance of local epidemiological data and susceptibility testing to guide antifungal therapy. These evolving resistance patterns reinforce the need for continuous surveillance and an individualized approach to antifungal selection in critically ill patients.

## Pharmacology of major antifungal agents

### Echinocandins, including caspofungin, micafungin, anidulafungin, and the recently introduced rezafungin, exhibit fungicidal activity against Candida spp. and are used as first-line treatment for invasive candidiasis. Their penetration into ocular, urinary, and CNS is poor and remains unclear into the peritoneal cavity

Echinocandins inhibit β-1,3-D-glucan synthesis, a key component of the fungal cell wall, and exert fungicidal activity against Candida spp. However, some species, such as C. parapsilosis, have higher minimum inhibitory concentrations (MICs) [43, 44]. The antifungal effect of echinocandins is concentration-dependent and characterised by a post-antifungal effect, making them the first-choice therapy for invasive candidiasis in critically ill patients [8]. First-generation echinocandins (caspofungin, micafungin, and anidulafungin) are administered parenterally once daily. The have high protein binding (> 95%) and good tissue distribution, particularly in the liver, kidneys, and lungs. However, ocular and CNS penetration is very limited, and urinary excretion is negligible [43]. Although hepatotoxicity and cardiotoxicity have been described with echinocandins in preclinical models [45, 46], the clinical impact of these findings is negligible. Conversely, the lack of clinically relevant drug-drug interactions may represent a significant advantage with the use of echinocandins [47]. Rezafungin, a new-generation echinocandin derived from anidulafungin, is characterised by an extended elimination half-life (> 130 h), allowing for once-weekly administration [41]. Phases II and III trials have demonstrated its non-inferiority to caspofungin in treating invasive candidiasis, leading to recent approval for this indication in Italy [48, 49].

Recommended dosing regimens for the different echinocandins are presented below:

- Caspofungin 70 mg loading dose at day 1 followed by a maintenance dose of 50 mg/day (dosing regimen should be increased to 100 mg loading dose followed by 70 mg maintenance dose in critical obese patients).
- Micafungin 100 mg/day maintenance dose with no loading dose.
- Anidulafungin 200 mg loading dose at day 1 followed by a maintenance dose of 100 mg/day.
- Rezafungin 400 mg loading dose at day 1 followed by a maintenance dose of 200 mg weekly.

### L-AmB has broad-spectrum fungicidal activity, including against Candida spp. In the treatment of invasive candidiasis, L-AmB is used in critically ill patients and intra-abdominal candidiasis as an alternative to echinocandins

L-AmB is an improved formulation of amphotericin B, encapsulated in a liposomal structure. This enhances its pharmacological properties since the liposoma acts as a sump while reducing the nephrotoxicity risk associated with older formulations. Its mechanism of action involves binding to fungal ergosterol with greater affinity than human cholesterol. In vitro, L-AmB exhibits fungicidal activity with a broad spectrum of action and a minimal risk of resistance development. L-AmB is administered exclusively intravenously in bound for ~ 95% to plasma proteins and reaches high tissue concentrations in the infected tissues thanks to the peculiar pharmacokinetic behaviour of the liposomal moiety [50]. Compared to conventional amphotericin B, L-AmB may be used at higher doses per kg without increasing the toxicity risk. Once absorbed, the reticuloendothelial system (RES) sequesters L-AmB, with the liver, kidneys, and lungs serving as primary reservoirs. L-AmB achieves low cerebrospinal fluid (CSF) penetration (~ 2–4% of serum levels), although this may reach up to 90% in children [51]. The standard dose (3 mg/kg/day) provides adequate intra-abdominal penetration, particularly in the presence of inflammation [45]. However, a recent trial suggested that a higher dose (5 mg/kg) may be helpful in preventing disease development in critically ill patients at high risk of intra-abdominal invasive candidiasis [52].

### In invasive candidiasis, azoles active against Candida spp., such as fluconazole and voriconazole, are primarily used in non-critically ill patients, as step-down therapy, and in the treatment of ocular and CNS infections

Azoles act by inhibiting ergosterol synthesis, a key component of the fungal cell membrane. Their activity is mainly fungistatic. Fluconazole is used for Candida infections, whereas triazoles extend their spectrum to include moulds but show variable activity against Candida spp. [53]. Azoles generally achieve excellent tissue penetration, though specific pharmacokinetic differences between agents may exist. For example:

- Fluconazole penetrates well into abdominal and CSF compartments.
- Voriconazole and isavuconazole shows high penetration into pulmonary, ocular, and cerebral tissues.
- Posaconazole has lower CNS penetration but are associated with fewer adverse effects than other azoles [43].

The main toxicity concern with azoles is hepatic dysfunction, which can manifest as elevated cytolytic and/or cholestatic markers. Additional side effects include the following:

- Voriconazole: visual disturbances and QTc prolongation.
- All azoles: significant drug interactions due to cytochrome P450 inhibition (strong for voriconazole, fluconazole, and posaconazole; mild-to-moderate for isavuconazole) [54].

For these reasons, fluconazole is commonly used as step-down therapy, including oral administration, for candidemia and intra-abdominal invasive candidiasis (see statement rationale 3.1) [4].

Recommended dosing regimens for the different azoles are reported below:

- Fluconazole: loading dose 12 mg/kg at day 1 followed by a maintenance dose of 6 mg/kg/day.
- Voriconazole: loading dose 6 mg/kg every 12 h at day 1 followed by a maintenance dose of 4 mg/kg every 12 h.
- Isavuconazole: loading dose 200 mg every 8 h in the first 48 h followed by a maintenance dose of 200 mg/day.

## Role of therapeutic drug monitoring (TDM), and the application of PK/PD targets

### In the ICU patient diagnosed with invasive candidiasis, the application of a TDM-guided strategy during fluconazole therapy, when indicated, may be helpful in preventing the risk of having a suboptimal PK/PD target related to pathophysiologic changes

Fluconazole is a hydrophilic drug primarily eliminated by the kidneys, and its plasma exposure can vary significantly in critically ill patients due to their specific pathophysiological conditions, potentially affecting the achievement of an optimal PK/PD target [36, 55]. Preclinical studies have identified the AUC/MIC (area under the curve/minimum inhibitory concentration) ratio as the best pharmacodynamic efficacy index for fluconazole. Specifically, an AUC/MIC ratio greater than 25–50 has been associated with maximal antifungal effect, even against Candida spp. expressing various resistance mechanisms [56]. A retrospective study of 77 critically ill, non-neutropenic patients with candidemia demonstrated that achieving an AUC/MIC ratio above 55.2 was associated with a significantly increased probability of survival (*p* = 0.008) [57]. In liver transplant patients, fluconazole penetration into the peritoneal and biliary compartments has been shown to be sufficient to reach the optimal PK/PD target [58]. Based on these findings, it has been proposed that implementing a TDM-guided strategy to maintain minimum plasma concentrations of fluconazole between 10–20 mg/L could maximise the achievement of an optimal PK/PD target in critically ill patients with abdominal-source ICI or post-liver transplantation [38, 58, 59]. This strategy may be particularly beneficial in high-risk subpopulations prone to underexposure with standard doses, such as patients with morbid obesity, augmented renal clearance, or those undergoing continuous renal replacement therapy (CRRT) [60, 61].

### Standard doses of echinocandins in critically ill patients with abdominal-source invasive candidiasis may result in insufficient drug exposure to achieve optimal PK/PD targets in peritoneal fluid, particularly against non-albicans Candida species. A TDM-guided dose adjustment might, therefore, be helpful.

Echinocandins are recommended as first-line therapy for abdominal-source IC according to international guidelines [4] However, their role in this setting has recently been debated following new evidence demonstrating poor penetration into peritoneal fluid, which increases the risk of suboptimal drug exposure and resistance selection, particularly in critically ill patients [62]. Caspofungin, micafungin, and anidulafungin exert a concentration-dependent antifungal effect, and the fAUC/MIC ratio has been identified as the best pharmacodynamic efficacy index in both preclinical and clinical studies (> 20 for C. albicans, > 7 for C. glabrata and C. parapsilosis). Clinical studies have shown that peak concentrations and AUC values for all the three echinocandins can be significantly lower in critically ill compared to non-critically ill patients and to healthy volunteers [63, 64]. Other studies have demonstrated that in patients with abdominal-source IC, the penetration of caspofungin, anidulafungin, and micafungin into ascitic fluid is limited, preventing the achievement of optimal PK/PD targets at the infection site and potentially favouring the selection of resistant strains [65–67]. As a result, several studies have highlighted the need for higher loading and maintenance doses of echinocandins in critically ill patients with abdominal-source ICI [68, 69]. Where TDM is unavailable, increasing standard doses should be considered an alternative approach to maximise the likelihood of achieving effective PK/PD targets, thereby reducing the risk of clinical failure and resistance selection. This strategy is particularly important in critically ill patients with physiological limitations that impair drug absorption or tissue distribution, such as those with peritoneal infections or septic shock [70].

### In critically ill patients with abdominal-source invasive candidiasis, there is insufficient evidence to support the clinical utility of a TDM-guided strategy for L-AmB in improving efficacy and/or tolerability.

L-AmB exhibits distinct PK/PD characteristics due to its unique pharmaceutical formulation, setting it apart from fluconazole and echinocandins in managing critically ill patients with abdominal-source IC. Its total plasma concentrations are significantly higher than those observed with amphotericin B deoxycholate, even when doses are normalised per kilogram of body weight. It is believed that most circulating drug is biologically inactive, with the liposomal formulation acting as a reservoir, releasing active drugs upon direct contact with the membrane of the fungus [71]. Unlike other antifungals, L-AmB has a predominantly non-renal and non-hepatic clearance, along with a prolonged elimination half-life, making it less susceptible to pathophysiological alterations commonly observed in critically ill patients [56, 62]. Preclinical studies have suggested that achieving a Cmax/MIC ratio > 10 may be associated with improved fungicidal activity. However, clinical evidence linking this PK/PD target to better efficacy remains limited [56]. A recent systematic review found no sufficient evidence to support the use of a TDM-guided strategy with Cmax/MIC targets of 25–50 for improving efficacy or AUC24h < 600 mg·h/L for minimising nephrotoxicity risk [72]. Consequently, implementing a TDM-guided strategy for L-AmB is currently neither recommended nor considered clinically useful.

### Limitations

This document has several limitations that should be acknowledged. First, it is not a GRADE-based document; therefore, clinicians should primarily rely on evidence-based clinical practice guidelines for decision-making on this topic. Second, no formal systematic review of the literature was performed, which may have introduced potential bias in the selection of supporting evidence. Third, the multidisciplinary nature of the panel may have broadened the perspective, which could sometimes dilute the specific focus on critically ill patients. Nonetheless, the inclusion of panelists with long-standing experience in this setting was intended to maintain the applicability of the recommendations to critically ill patients.

## Conclusions

Invasive candidiasis are challenging infections in critically ill patients, where non-specific risk factors, diagnostic challenges, and variable pharmacokinetic profiles of antifungal agents often hamper optimal management. Timely initiation of appropriate antifungal therapy, guided by clinical risk assessment, infection site characteristics, and PK/PD considerations, can help improving overall appropriateness of antifungal management of invasive candidiasis in critically ill patients.

## Supplementary Information

Additional file 1: Full reporting of the voting process, per original statement.

Additional file 2.

## Data Availability

Not applicable.

## Abbreviations

AUC/MIC: Area under the curve/minimum inhibitory concentration
BDG: Beta-D-glucan
CNS: Central nervous system
CSF: Cerebro-spinal fluid
CRRT: Continuous renal replacement therapy
IC: Invasive candidiasis
ICU: Intensive care unit
L-AmB: Liposomal amphotericin B
PK/PD: Pharmacokinetic and pharmacodynamic
TDM: Therapeutic drug monitoring
