# Supplementary material for: Diagnosis and management of invasive candidiasis in critically ill patients: SIAARTI multidisciplinary statement
Source: J Anesth Analg Crit Care. 2025 Dec 2;6:1. doi: 10.1186/s44158-025-00299-y (PMC12777504; doi:10.1186/s44158-025-00299-y)
Supplement: Supplementary file 2 — Additional file 2. [file 44158_2025_299_MOESM2_ESM.docx]

## Annex 1 – Voting of the statements and rationale
